# The albumin-binding domain as a scaffold for protein engineering

**DOI:** 10.5936/csbj.201303009

**Published:** 2013-09-01

**Authors:** Johan Nilvebrant, Sophia Hober

**Affiliations:** a Division of Protein Technology, School of Biotechnology, KTH Royal Institute of Technology, AlbaNova University Center, SE-106 91 Stockholm, Sweden

**Keywords:** protein domain, albumin, specificity, affinity protein, affinity maturation, bispecific

## Abstract

The albumin-binding domain is a small, three-helical protein domain found in various surface proteins expressed by gram-positive bacteria. Albumin binding is important in bacterial pathogenesis and several homologous domains have been identified. Such albumin-binding regions have been used for protein purification or immobilization. Moreover, improvement of the pharmacokinetics, through the non-covalent association to albumin, by fusing such domains to therapeutic proteins has been shown to be successful. Domains derived from streptococcal protein G and protein PAB from *Finegoldia magna*, which share a common origin and therefore represent an interesting evolutionary system, have been thoroughly studied structurally and functionally. Their albumin-binding sites have been mapped and these domains form the basis for a wide range of protein engineering approaches. By substitution-mutagenesis they have been engineered to achieve a broader specificity, an increased stability or an improved binding affinity, respectively. Furthermore, novel binding sites have been incorporated either by replacing the original albumin-binding surface, or by complementing it with a novel interaction interface. Combinatorial protein libraries, where several residues have been randomized simultaneously, have generated a large number of new variants with desired binding characteristics. The albumin-binding domain has also been utilized to explore the relationship between three-dimensional structure and amino acid sequence. Proteins with latent structural information built into their sequence, where a single amino acid substitution shifts the equilibrium in favor of a different fold with a new function, have been designed. Altogether, these examples illustrate the versatility of the albumin-binding domain as a scaffold for protein engineering.

## Introduction

Many gram-positive bacteria express surface proteins with ability to bind serum proteins [1]. The surface proteins typically contain tandemly repeated serum protein-binding domains with one or several specificities, which often include albumin binding [2, 3]. The bacteria can thereby camouflage themselves with bound host-proteins to evade the immune system and potentially also scavenge protein-bound nutrients [4, 5]. Albumin is the most abundant protein in plasma and expression of albumin-binding proteins has been shown to promote bacterial growth and virulence [5, 6]. The bacterial species that express albumin-binding domains are usually part of the normal human flora and they are opportunistic pathogens. There are many different types of albumin-binding proteins with different size and function. For example, more than 40 albumin-binding domains have been found in one protein, forming a rod-like structure in a giant cell wall-associated fibronectin-binding molecule. This protein was found on the surface of *Staphylococcus aureus* and is called Ebh (ECM-binding protein homologue, Uniprot Q2FYJ6) [7, 8]. These huge proteins, which have also been found on streptococci (i.e. extracellular matrix-binding protein (Embp), Uniprot Q8KQ73) [9], are in addition able to bind fibronectin. They mediate adhesion and have been shown to be required for biofilm formation *in vivo*. An additional mechanism of albumin binding was recently identified when it was shown that human serum albumin (HSA) adsorbed to bacteria could bind to and inactivate the antibacterial chemokine MIG/CXCL9 (monokine-induced by gamma-interferon/CXC ligand), which is released by activated epithelium [10]. This albumin-dependent event protects from the antibacterial activity and promotes bacterial survival at the epithelium. Even though all functions of bacterial surface proteins are not yet fully elucidated, they clearly provide the bacteria expressing them with an evolutionary advantage.

**Figure 1:**
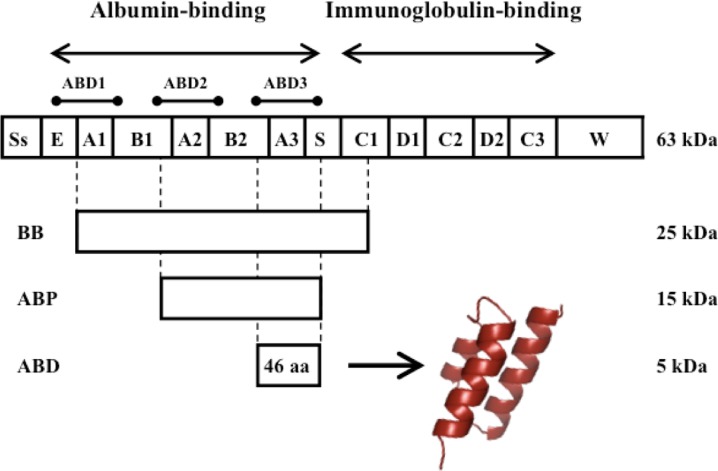
**Schematic representation of streptococcal protein G**. Protein G consists of an N-terminal signal sequence (Ss), an albumin-binding region containing three albumin-binding domains and a C-terminal immunoglobulin-binding region. A spacer (S) separates the binding regions and a C-terminal sequence (W) anchors the protein to the cell wall. Various albumin-binding parts, BB, ABP and the smallest albumin-binding unit, the 46 amino acid albumin-binding domain (G148-ABD), are indicated. ABD folds into a stable three-helix bundle structure (the picture was generated from PDB-file 1GJT).

Streptococcal protein G (SPG), which binds to immunoglobulins and albumins of several species, is expressed on the surface of certain streptococcal strains [11–13] and is one of the best-characterized bacterial surface proteins. As indicated in Figure 1, SPG from the opportunistic streptococcal strain G148 has two functional regions containing three immunoglobulin-binding (C1-C3) and three albumin-binding domains (ABD1-3), respectively [12, 14]. The immunoglobulin-binding domains share a common four-stranded beta-sheet fold with a single alpha helix packed onto the sheet (4ß + α) [15]. Of the three homologous albumin-binding domains, the C-terminal ABD3 has been most extensively studied; it is referred to as G148-ABD in the text and G148-ABD3 in Figure 2A. Nuclear magnetic resonance (NMR) spectroscopy has established that this 46 amino acid domain folds into a left-handed anti-parallel three-helix bundle (3α) [4, 16], similar to the structure of the immunoglobulin-binding domains of the well-studied staphylococcal protein A [17, 18]. This structural element is found in several other proteins, which indicates that the 3α-fold is energetically and functionally favorable since it has been utilized broadly [19]. Interestingly, a structural evaluation of the repeating units in the giant albumin-binding protein Ebh showed that its domains, one of which is responsible for albumin binding, are connected by a long helix that participates in two three helix bundles in two adjacent repeating units [8]. This helix is responsible for the global rod-like structure of the protein.

**Figure 2A:**
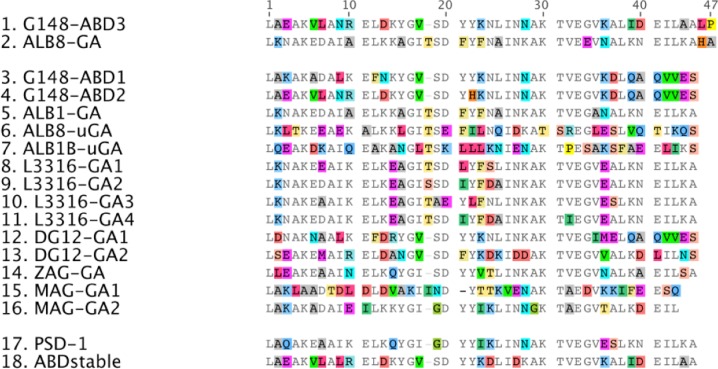
**Sequence alignment of 16 homologous albumin-binding domains and two engineered variants**. Conserved amino acids are shown in gray and differences are highlighted in color. G148-ABD3 and ALB8-GA (sequences 1 and 2) represent the best-studied domains. PSD-1 (sequence 17) is an engineered variant with broadened species specificity and ABDstable (sequence 18) is a variant that has been stabilized to alkaline treatment. The picture was generated in Geneious Pro version 5.5.7 created by Biomatters and is based on a similar picture by Johansson et al. [4].

Historically, the most widespread use of SPG has been as a biotechnological tool mainly used for affinity purification of immunoglobulins exploiting the broad species- and subclass specificity of its immunoglobulin-binding domains [20, 21]. Albumin-binding regions spanning one or several albumin-binding domains, for example BB and ABP [22] that are indicated in Figure 1, have been used for affinity purification or depletion of albumin [21]. Moreover, the use of an albumin-binding region as a fusion tag can facilitate affinity purification of a target protein, improve its solubility or be used for directed immobilization [22–24].

Several homologous albumin-binding domains have been identified in surface proteins from different bacterial species. The sequence diversity among these is illustrated by the 16 homologues included in Figure 2A. Alongside G148-ABD, the so-called protein G-related albumin-binding (GA) module from protein PAB (peptostreptococcal albumin-binding) of the anaerobic bacterium *Finegoldia magna* (*F. magna*) has been thoroughly investigated both structurally and functionally [19, 25, 26]. Analysis of the gene encoding PAB suggested that its albumin-binding domain (ALB8-GA representing the best characterized variant, see Figure 2A) originates from protein G and that it was introduced as a result of an interspecies module-shuffling event [25]. Available data on various albumin-binding domains suggest a correlation between the species specificity of the surface proteins and the host specificity of the bacteria that express them [4]. G148-ABD and ALB8-GA exhibit 59% amino acid sequence identity, but the species specificity of G148-ABD is much broader than for ALB8-GA whereas the binding affinity of ALB8-GA for HSA is roughly twofold higher. ALB8-GA has only been found in human isolates of *F. magna* and, consequently, it is believed to have evolved to bind HSA with higher affinity than its predecessor. In contrast, streptococci expressing G148-ABD have much broader host specificity and this domain binds albumin from several non-primates better than ALB8-GA [4].

Accumulated structural data on G148-ABD [4, 16] and the GA-module [26–29] demonstrate that the domains have very similar tertiary structures. ALB8-GA contains an additional residue in the loop between the first and second helix (Figure 2A) and has a somewhat shorter first helix compared to G148-ABD [4]. The lengths and positions of the second and third helices are almost identical and this region also contains the most highly conserved sequence stretch among the homologues (Figure 2A), which implies that they all share a common overall fold. As would be expected, competitive binding studies have shown that G148-ABD and ALB8-GA have the same binding site on HSA [4]. A crystal structure of ALB8-GA in complex with HSA revealed that this site is located on the exterior of domain II of the albumin molecule [28], Figure 2B. The flat binding site consists of a hydrophobic center and two surrounding hydrogen bond networks [28]. A similar structural complex of ALB8-GA and a fatty acid-induced conformational form of HSA demonstrated that both forms could be recognized [29]. Mainly residues in the second helix and the following loop of G148-ABD contribute to albumin binding, as determined by a dedicated mutational study [30].

**Figure 2B:**
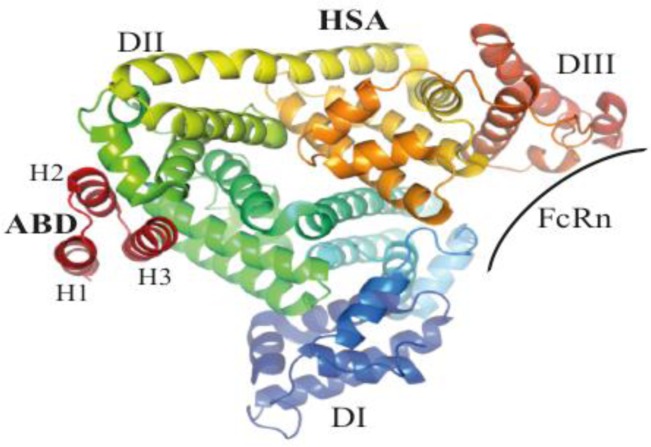
**Structure of the complex formed by ALB8-GA and HSA**. The albumin-binding domains recognize a site located in domain II of HSA that does not overlap with the binding site for the neonatal Fc-receptor (FcRn), which plays an important role in albumin homeostasis. The picture was generated from PDB-file 1TF0.

To localize the binding site, surface exposed residues or combinations of residues pointing in different directions have been substituted with alanine and subjected to a binding analysis to HSA and an evaluation of secondary structure content by circular dichroism spectroscopy [30]. In the next step, several single residues as well as combinations of residues in the proximity of a functionally important amino acid, Tyr21 located in the second helix (Figure 2A, all numbering in the text is based on the numbering in this figure), were substituted. The corresponding variants were analyzed to determine the binding contributions of each residue relative the wild-type variant and thereby define the binding site. The most important residues were found to reside in the second helix and in the loop to the third helix. This study demonstrated that the binding of G148-ABD to HSA can be abolished by only a few amino acid changes and the overall mapped binding region in G148-ABD is largely supported by NMR-perturbation studies performed on both the homologous ALB8-GA [26] and G148-ABD [4] and by the ALB8-GA:HSA structural complex [28]. However, the NMR-studies generally assign larger binding surfaces, which may in part be due to contacts between the albumin-binding domains, as indicated by the crystal structure of a dimer of ALB8-GA [27]. Neither NMR nor X-ray studies have specified the central importance of the second helix for binding as accurately as the mutational analysis of G148-ABD.

## Protein engineering of ABD

**Figure 3:**
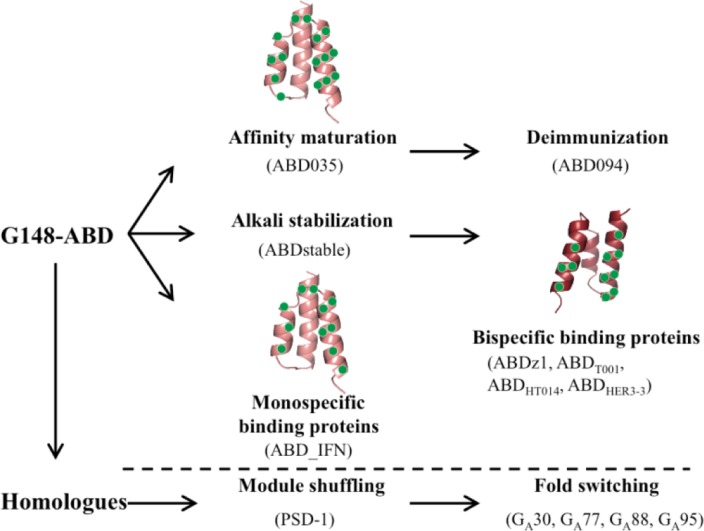
**Engineered albumin-binding domains**. Several engineered domains have been constructed based on G148-ABD or through shuffling of a set of homologous sequences. Variants mentioned in the text are shown together with a structural representation of the residues that have been randomized in three different combinatorial libraries (based on PDB-file 1GJT).

Both G148-ABD and ALB8-GA are, despite their small size, very stable domains in themselves, without any additional stabilizing features such as bound ions or disulfide bridges. A compact hydrophobic core has been suggested to be responsible for the high melting temperature and the high tolerance to both high and low pH and treatment with guanidium hydrochloride [19, 31, 32]. Other attractive characteristics include a high solubility and expression level and an ability to refold after thermal or chemical denaturation. The small size also makes the domain amendable to peptide synthesis. These features make this three-helix bundle domain a suitable scaffold for further protein engineering efforts (Figure 3). Both rational and combinatorial approaches have been used where mutants are either screened individually or in large combinatorial libraries using *in vitro* selection systems such as phage display. Similar efforts, for example using the structurally related Z-domain [33] as a scaffold, have demonstrated the potential of this approach to provide molecules with new and/or improved characteristics [21].

## Engineering of ABD to understand species specificity

The well-defined sequence space that the albumin-binding domains represent offers an opportunity to address sequence determinants for their natural phenotypic variations. It has been proposed that a phenylalanine in position 21 of ALB8-GA (Figure 2A) is responsible for its high affinity and specificity for HSA and other primate albumins, which would be mediated through an interaction with the hydrophobic Met329 in these albumins [28]. The corresponding tyrosine residue in G148-ABD can potentially interact more broadly with various polar or charged amino acids on albumins from different species. In an effort to understand such determinants for species specificity, a protein engineering approach called offset recombinant polymerase chain reaction (PCR) [34] was used to shuffle homologous albumin-binding sequences [34]. Seven so-called template domains were designed by introducing point mutations in G148-ABD based on the sequences of native albumin-binding domains. Shuffling of these template sequences and subsequent cloning into a phage display vector generated a library that was screened for binders to HSA, guinea pig serum albumin (GPSA) or both targets in alternate rounds of selection. HSA and GPSA were selected as targets because ALB8-GA has a 1000-fold preference for binding HSA over GPSA whereas G148-ABD binds both forms with similar affinities [4]. In addition, the targets represent opposite ends of a phylogenetic tree of albumins from different species and GPSA contains a polar threonine residue where HSA has the non-polar methionine 329 [34].

Surprisingly, all selection strategies showed a clear preference for the same variant, called phage-selected domain 1 (PSD-1) (Figure 2A). PSD-1 is more similar to G148-GA than ALB8-GA on the sequence level and retains the Tyr21 of G148-ABD, which may partly explain its broad specificity. Another interesting feature of PSD-1 is the introduction of a lysine in position 39 (Figure 2A), a characteristic that is shared with ALB8-GA and also commonly found among the homologues. An NMR-structure of PSD-1 showed that this substitution stabilized the backbone in a conformation consistent with the albumin-bound ALB8-GA [35]. The resulting closer packing of the third helix in the core of PSD-1 may also explain its higher melting temperature (85°C) compared to G148-ABD (75°C), which has an isoleucine in this position [34]. Data on the dynamics of PSD-1 also demonstrate that, since PSD-1 is less flexible than G148-ABD and at the same time binds phylogenetically diverse albumins more tightly, broad species specificity can be achieved without an increased backbone flexibility [35]. Previous studies have proposed that the backbone flexibility of G148-ABD is the reason behind its broader specificity compared to ALB8-GA [4]. Consequently, polymorphism at position 21 offers a more likely mechanism for albumin specificity and, in the absence of PSD-1, the relative contributions of the tyrosine in the binding interface and the backbone dynamics were difficult to asses since both features were present in G148-ABD and absent in ALB8-GA [35]. To further analyze the mechanism of the broad specificity of the rigid PSD-1, its binding to a range of albumins was mapped using chemical shift perturbation [36]. These data support the mutational mapping [30] and imply that the contacts along the entire length of the third helix are not as important as indicated by the crystal complex [28]. However, small displacements of the third helix may lead to changes in albumin affinity that influence the specificity, even though PSD-1 uses essentially the same binding epitope to interact with phylogenetically diverse albumins [35, 36].

## ABD and serum half-life

Not only bacteria can benefit from albumin binding, for example a large number of studies have demonstrated its potential as a mean to achieve longer half-lives of therapeutic proteins [37]. Albumin has an extraordinarily long circulatory half-life of 19 days in humans as a result of a size above the renal filtration cutoff and a pH-dependent binding to the neonatal Fc-receptor (FcRn), which provides a rescue mechanism to divert albumin and immunoglobulin G (IgG) from a lysosomal degradation pathway [38]. As a consequence, non-covalent association to albumin can be used to extend the half-life of drugs, which has been investigated using several albumin-binding molecules including G148-ABD [39, 40]. Importantly, the FcRn-binding site on albumin is located in domain III [41] and does not overlap or interfere with binding to G148-ABD [42, 43] (Figure 2B).

**Figure 4:**
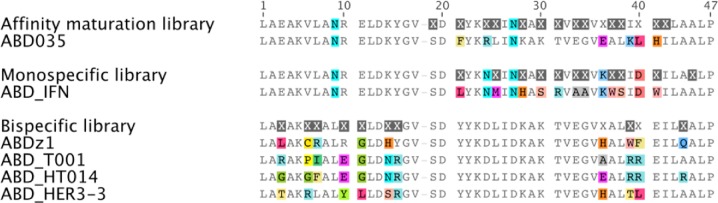
**Combinatorial protein libraries based on G148-ABD and selected variants from them.** Sequence alignment of three combinatorial libraries based on G148-ABD and examples of variants originating from them. Common residues are shown in gray and differences in color, X indicates randomized positions regardless of the degree or type of diversification used in the library design. The figure was generated with Geneious Pro version 5.5.7.

Inspired by the promising features of G148-ABD as a half-life prolonging fusion partner for protein therapeutics, it has been subjected to affinity maturation for HSA to enable further improvements of the pharmacokinetics [44]. In this effort, 15 residues in helices two and three (Figures 3 and 4) were diversified followed by library selection against HSA by phage display. The choices of positions and randomization schemes were based on sequences of homologues, available structural data of G148-ABD and ALB8-GA and their albumin-binding residues. Two libraries were pooled to account for the variability caused by the extra amino acid in the first loop of ALB8-GA compared to G148-ABD. Sequencing revealed that, in as many as nine of the 15 varied positions, the wild-type residue occurred in a majority of the selected clones. Interestingly, none of the selected variants originated from the sub-library containing the additional residue in the first loop. Based on data from the first generation of variants, seven new domains were rationally constructed to share a common C-terminal segment. One of these new variants, ABD035 (Figure 4), had an extremely high affinity with an equilibrium dissociation constant (K_D_) for HSA of 120 fM [39], improved binding to albumin of several other species and beneficial biophysical properties [44]. ABD035, which differs from G148-ABD in seven positions, has several interesting sequence characteristics that can be related to previous studies of albumin-binding domains. First, the preference for phenylalanine instead of tyrosine in position 21 correlates with the suggested importance of this residue for strong binding to HSA [28]. A beneficial spontaneous substitution at a position not variegated in the library design (I39K) was found in two clones and was also included in all the second-generation variants. Interestingly, the recombined albumin-binding domain PSD-1 [34] described above contains the same substitution (Figure 2A) and other variants in the affinity maturation study contained I39T substitutions, which indicates that substitution of this position can be beneficial for binding. Surprisingly, all second-generation variants except ABD035 were prone to aggregation. The high solubility of ABD035 is presumably due to a unique arginine residue in position 24 (Figure 4). Altogether, the more than 2000-fold improved affinity for albumin seems to be a result of an optimization of both the composition of surface exposed residues and the structural conformation. For example, the melting temperature of ABD035 was higher than for a selected first generation variant but still significantly lower compared to G148-ABD, which indicates that the improved affinity does not solely result from lower entropy of the binding. However, a thorough structural characterization of ABD035 is necessary to fully resolve such molecular details.

Both G148-ABD and ABD035 have successfully been evaluated as half-life extending fusion partners *in vivo* to achieve significantly improved pharmacokinetics of the protein of interest [39, 45]. Interestingly, a side-to-side comparison of ABD035, the wild-type G148-ABD and a weakly binding variant (G148-ABD_Y22A_, [30]); representing affinities of 120 fM, 5 nM and 330 nM for HSA, respectively, and all within a span from 2-600 nM for mouse serum albumin, indicated that improved half-life could be achieved also from weak association to albumin [46]. This has also been shown previously by using peptides with weak albumin-binding affinities [47]. However, a study using a very low affinity variant of G148-ABD (G148-ABD_S19A, Y21A, K23A_; [30] demonstrated that its affinity was below the threshold necessary to achieve a half-life extension [39]. The bacterial origin of the albumin-binding domain raises concerns regarding its immunogenicity, yet the bacterial proteins have evolved to mediate immune escape. Nevertheless, ABD035 has been subjected to a deimmunization strategy by substituting residues in immunogenic regions while maintaining the high albumin binding affinity and favorable biophysical characteristics [39]. Assays comparing a series of deimmunized variants identified a candidate, denoted ABD094, which in contrast to G148-ABD or ABD035 had no immunogenic potential in T-cell proliferation assays, where it was as inert as the control HSA. ABD094 is currently in multiple development programs (Affibody AB, unpublished data).

## Stabilization of ABD

To improve its properties as an affinity ligand for purification or depletion of albumin, G148-ABD has been engineered for improved tolerance to alkaline conditions to withstand harsh cleaning of chromatographic equipment [31]. A straightforward protein engineering strategy, based on substituting asparagine residues that are susceptible to base-catalyzed deamidation with amino acids found in homologous sequences, resulted in a new molecule, ABDstable (Figure 2A), with a dramatically improved stability to repeated alkaline exposure. Replacement of a total of four asparagine residues (N9L, N24D, N27D and N28K) at the same time improved the stability to chemical and thermal denaturation compared to G148-ABD. The introduction of a hydrophobic residue at a position in the first helix that points inwards is most likely responsible for the improved thermal stability (+10°C) whereas the remaining modified residues are surface exposed and unlikely to promote such effects [31]. Construction of a dimeric molecule with a stabilized linker sequence led to further improvements in alkaline stability and chromatographic performance [48].

## Engineering new binding sites into albumin-binding domains

Novel binding sites can be engineered into a protein domain to achieve a desired molecular recognition function while retaining the favorable biophysical properties of the scaffold protein. The most widespread three-helical protein scaffold is the Z-domain, in which the inherent immunoglobulin-binding site has been randomized to generate libraries of so called Affibody molecules that can be selected to bind a wide range of target proteins and provide affinity proteins for various applications [49]. Another similar three-helical scaffold that has been used for library constructions and selections is the *Measles* virus phosphoprotein P, which is a stable protein framework that was identified based on its structural similarity to the Z-domain and its encouraging physiochemical properties [50].

G148-ABD has been used as a scaffold to generate both mono-[51] and bispecific [52] affinity proteins by randomization of the albumin-binding surface or a surface located on the opposite face of the molecule, respectively (Figures 3 and 4). To substitute the albumin-binding surface with a new binding site, eleven residues were identified as suitable for diversification using various *in silico* methods [51]. These residues, distributed over the last two helices and their interconnecting loop (Figure 4), were randomized and the library was screened for binders to interferon-γ using ribosome display. The selected molecules recognized the new target with low nanomolar affinities and did not have any residual binding to albumin or other unrelated control proteins in an enzyme-linked immunosorbent assay. Ten of the residues targeted for mutagenesis, all except A45, were also diversified during the affinity maturation of G148-ABD [44], (Figure 4) and none of them reverted to the wild-type residue after selection of binders to interferon-γ, which indicates that these substitutions were well tolerated. Of the additional five residues that were diversified by Jonsson et al., one was considered non-mutable (I42) and the potential for diversifying the remaining four positions (S19, N24, K36 and D40) was not discussed further. Interestingly, three of these residues reverted to wild type in a majority of the clones found after the affinity maturation of G148-ABD (S19, N24 and I42) whereas K36 and D40 were substituted from wild type in several affinity-matured variants including ABD035 (Figure 4). However, more data on the contribution of each residue to the binding and stability of the new binding molecules are required to assess the general applicability of this approach.

A more challenging approach was aimed at incorporating a novel binding site in G148-ABD while retaining the inherent albumin-binding ability, thus resulting in 46 amino acid bispecific protein domains. The mutational mapping of the albumin-binding site in G148-ABD [30] suggested that as many as nine residues on the surface of the first and third helix could be substituted without any significant loss of structure, stability or HSA-binding ability. These residues, plus two additional surface exposed positions that displayed natural variation in the homologues (Y15 and A44; Figure 4), were randomized using the stabilized variant ABDstable [31] as a scaffold. Two of the total eleven randomized positions (K36 and D40 in the third helix) were also randomized in the G148-ABD affinity maturation library [44] and, while several of the remaining positions vary between homologues, none of them has been diversified in other combinatorial libraries previously. Analysis of phage stocks from selections against HSA by Western blot showed that albumin binding could be retained in the library despite the high degree of substitutions in the two helices [52]. The library was next subjected to selection against a dimeric form of the Protein A-derived Z-domain [33]. This selection identified one variant, ABDz1 (Figure 4), with acquired affinity for Z (an apparent affinity of 0.4 µM) and retained binding to HSA. ABDz1 contains a cysteine in the beginning of its first helix and subsequent experiments showed that the Z-binding was disrupted when this residue was replaced with a serine or when a reducing agent was present. Moreover, head-to-tail dimers of ABDz1_C6S_ did not bind Z and, consequently, ABDz1 could only bind its target when present in a disulfide-bridged dimeric conformation. Utilizing its small size and dual binding specificities, ABDz1 has been used as an affinity fusion tag for an effective orthogonal affinity purification strategy [52, 53].

The bispecific library has also been screened for binders to tumor necrosis factor alpha (TNF-α) [54]. An initial phage display selection identified two bispecific variants, one of which bound TNF-α with a moderate affinity (385 nM apparent affinity) and HSA only weakly (1.9 µM) while the other variant bound strongly to HSA (17 nM) but only weakly to TNF-α (1.6 µM). These results pointed toward the challenge of obtaining two high affinity interactions in the same small protein domain. To explore this subject further, an affinity maturation library was designed based on the sequences of the two initial hits. Eight of the initial eleven residues were re-randomized and the library was expressed and displayed on the surface of staphylococcal cells to allow for multi-parameter fluorescence-activated cell sorting. In these selections the library was screened for binders to TNF-α and, in a parallel selection, cells binding both TNF-α and HSA in the same sorting cycle were enriched using an additional fluorescent label. Characterization of variants from both selection strategies, illustrated by ABD_T001_ selected against TNF-α and ABD_HT014_ selected against TNF-α and HSA simultaneously (Figure 4), demonstrated that affinities in the nanomolar range for both targets could be achieved (apparent affinities of 3-5 nM for TNF-α and K_D_ down to 35 nM for HSA) and that the affinity maturation resulted in a roughly 100-fold improved TNF-α-binding. An interesting finding in the variants selected for TNF-α and HSA was a common, charge-switching, K36E substitution that was also observed during affinity maturation of G148-ABD for HSA [44]. However, none of the diversified residues reverted to the wild-type amino acid in any of the common clones identified after the dual selections.

In an effort to expand the single domain bispecific concept to a cancer-related target protein, binders to the epidermal growth factor receptor 3 (HER3) have been selected [55]. In contrast to previous selections, phage display panning on this target generated more than 30 unique but highly similar variants, represented by the strongest binder ABD_HER3-3_ in Figure 4. All analyzed candidates bound HER3 with affinities in the nanomolar range and all retained a strong, or even improved, binding to HSA (for example, ABD_HER3-3_ binds HER3 with a K_D_ of 10 nM and HSA with 0.4 nM). The binding site on HER3 was shown to overlap with its ligand-binding site, indicating a potential anti-proliferative effect on HER3-overexpressing cells. Currently, variants with sub-nanomolar affinity for both their target protein and HSA are under development (unpublished data). Together, these selections demonstrate that albumin binding can be retained despite randomization of a large number of residues in helix one and three. Up till now no binders with ability to bind albumin and the target simultaneously have been identified and no selections have been designed to address this property. Presumably, simultaneous binding would require a specific geometry of the target and the binding epitope in relation to the albumin-binding site, which has not yet been fulfilled for the currently evaluated target proteins.

## Albumin-binding domains in folding studies

**Figure 5:**
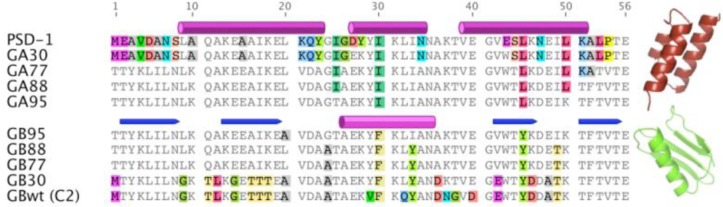
**Sequence alignment of very similar variants with different tertiary structures.** Identical residues are shown in gray and differences are highlighted in color. The top five sequences form three-helix bundles (illustrated using the PDB-file 1GJT) and the lower five sequences form 4ß + α folds (PDB-file 1FCC). Elements of secondary structure are indicated above the sequences. The figure is based on an alignment in Shen et al. [65] and was generated with Geneious Pro version 5.5.7.

Albumin-binding domains and other small domains have proven to be attractive model systems for experimental and theoretical folding studies [16, 56, 57]. For example, studies of engineered proteins that can switch folds provide insights into how new folds evolve and also demonstrate that latent structural information can be encoded in an amino acid sequence. Directed evolution of the immunoglobulin-binding domains of SPG and staphylococcal protein A has been used to generate pairs of monomeric proteins with a high degree of sequence identity but with different tertiary structures [58, 59]. A similar effort has subsequently been devoted to designing pairs of proteins with very high sequence similarity, different folds and also different binding functions. Here, the starting points were 56 residue polypeptides encoding non-homologous albumin- and immunoglobulin binding domains derived from SPG [60] (Figure 5). The albumin-binding domain used in this study was PSD-1 [34], the additional residues required to form the immunoglobulin-binding fold are located in the termini and are disordered in the 3α-fold of PSD-1. The first step in the process of making the two proteins more similar to each other was to encode latent binding sites for IgG in PSD-1 and for HSA in the 4ß + α IgG-binding protein, while preserving their structures and original binding functions. This resulted in a pair of proteins with 30% identity, the albumin-binding G_A_30 and the immunoglobulin-binding G_B_30 (Figure 5). Next, the binary sequence space that separated these two sequences was reduced in a step-wise manner to generate variants of even higher sequence identity. Pairs with 77% and 88% identity were generated and NMR-analysis showed that the two folds were retained in all four variants (i.e. G_A_77 and G_B_77 or G_A_88 and G_B_88; Figure 5). The IgG-binding was functional only in the 4ß + α fold and the HSA-binding only in the 3α-fold. Thus, this protein engineering endeavor demonstrated that as few as seven residues that differed between G_A_88 and G_B_88 could determine both the fold and function of the domains.

Analysis of the geometries of the non-identical residues in NMR-structures of G_A_88 and G_B_88 facilitated the design of a new pair of sequences with an impressive 95% identity (G_A_95 and G_B_95; Figure 5) [61]. Structures of G_A_95 and G_B_95, which only differ in three positions, demonstrated that a single amino acid substitution could cause a conformational switch between the two functionally diverse folds [62]. A following study determined the NMR-structures of a series of variants, which only differed by one amino acid, and identified three mutational tipping points (L20A, T25I and L45Y) that shifted the equilibrium between the two possible folds [63]. These studies illustrate the exceptional mutational tolerance of the albumin-binding domains. Moreover, the data form the basis for a plausible hypothesis regarding the evolution of new protein structures and functions. Perhaps a duplicated albumin-binding domain acquired the immunoglobulin-binding fold in the multi-domain bacterial surface protein, where the multiple domain copies could allow the evolution of such gain of functionality without any significant loss of fitness [63, 64].

## Summary and outlook

Despite the small size, albumin-binding domains have successfully been engineered for several purposes. To date, more than a hundred engineered variants with altered specificity, improved affinity or stability and even new binding specificities have been reported. Large libraries of domains with diversified surface patches or shuffled homologous sequences have been displayed on phages, on ribosomes and on the surfaces of bacterial cells to facilitate *in vitro* selection of desired variants. Even though more than 50% of the residues have been substituted in these efforts, many generated variants retain the favorable fold and stability of the original domain. Several beneficial sequence modifications have been discovered and structurally important residues that cannot easily be modified have been identified. The current detailed understanding of this defined sequence space provides a basis for further protein engineering efforts. Promising characteristics found in engineered domains might be combined and structural characterization of more variants would be useful for future efforts in this direction. Clever protein engineering strategies applied to a small protein domain with favorable biophysical properties such as the albumin-binding domain provides many exciting future opportunities for biophysicists or bioinformaticians engaged in the relationships between sequence, structure and function of proteins, as well as for protein engineers interested in new therapeutic applications.
